# The Changing Epidemiology of Hepatitis C Virus Acquisition Among HIV-Infected Individuals in Brazil

**DOI:** 10.1089/aid.2021.0197

**Published:** 2023-01-11

**Authors:** Rosario Quiroga Ferrufino, Ana Luiza Bierrenbach, Camila Rodrigues, Gerusa Maria Figueiredo, Daniel Gleison, Silvia Yapura, Maria Laura Mariano de Matos, Ricardo Vasconcelos, Steven Sol Witkin, Maria Cássia Mendes-Correa

**Affiliations:** ^1^ Faculdade de Medicina, Departamento de Molestias Infecciosas e Parasitarias, Universidade de São Paulo, Sao Paulo, Brazil.; ^2^ Instituto de Ensino e Pesquisa, Hospital Sírio-Libanês, Sao Paulo, Brazil.; ^3^ Faculdade de Medicina, Hospital das Clínicas, Universidade de Sao Paulo, Sao Paulo, Brazil.; ^4^ Faculdade de Medicina, Instituto de Medicina Tropical, Universidade de São Paulo, São Paulo, Brazil.; ^5^ Faculdade de Medicina, Departamento de Medicina Preventiva, Universidade de São Paulo, Sao Paulo, Brazil.; ^6^ Department of Obstetrics and Gynecology, Weill Cornell Medicine, New York, New York, USA.

**Keywords:** hepatitis C, HIV, coinfection, epidemiology, transmission

## Abstract

Identification of mechanisms of hepatitis C virus (HCV) acquisition among HIV-infected people is critical for prevention guidance. The aim of this study was to investigate risk factors for HCV infection and variations in HCV genotype distribution in a cohort of HIV-HCV coinfected patients in Brazil. This was a cross-sectional observational epidemiological study of a cohort of HIV-HCV coinfected individuals seen at a referral center for HIV-infected patients in the city of São Paulo between January and December 2017. The time of HCV acquisition, as determined by chart review, was categorized as before 2000, between 2000 and 2009, and from 2010 onward. HCV genotypes were determined by gene amplification and analysis. Among 3,143 HIV-infected individuals analyzed, 362 (11.5%) were HCV-HIV coinfected. Overall, the reported modes of HCV acquisition were sexual exposure in 172 (47.5%), injection drug use (IDU) in 86 (23.8%), use of inhaled drugs in 67 (18.5%) and blood transfusion in 10 (2.8%) individuals. All individuals who acquired HCV after IDU became infected before 2010. HCV acquisition by sexual contact was reported by 26.4%, 65.9%, and 63.8% of patients before 2000, between 2000 and 2009, and from 2010, respectively. There was an increase (*p* < .001) in the proportion of cases due to sexual transmission from the period before 2000 (26.4%) to between 2000 and 2009 (65.9%). There was no corresponding increase from 2000 and 2009 to after 2010 (*p* = .751). HCV genotype 1 was most prevalent at all time periods. The genotype 3 frequency decreased over time (test for trend *p* < .001), whereas genotype 4, extremely uncommon before 2010, became the second most prevalent genotype from 2010 onward. In HIV-infected individuals in Sao Paulo, Brazil, sexual transmission has replaced IDU as the most frequent mode of HCV acquisition.

## Introduction

According to data from the World Health Organization (WHO), of the 36.7 million people living with HIV, 2.3 million are also infected with hepatitis C virus (HCV).^1^ Several studies published up to the year 2000 indicated that HCV-HIV coinfection occurred most frequently in injection drug user (IDU) and HIV-infected hemophiliac patients.^2^ Sexual transmission of HCV between men who have sex with men (MSM) and heterosexuals living with HIV who were not IDU, although mentioned, was not considered an important mechanism in the epidemiological chain of HCV transmission.^2–4^ Since 2000, there has been a marked increase in the prevalence of acute HCV infection in HIV-positive MSM in Europe, the USA, Australia,^5–9^ and HCV infection in this group has emerged as a worldwide epidemic.^10^ However, according to the WHO, IDU still is the most frequent mechanism of exposure to HCV, reaching up to 1.36 million people (82.4% of HCV-HIV coinfections) followed by MSM in 6.4% of cases.^1^

In contrast to the situation in other parts of the world, only very limited recent data are available concerning the epidemiology of HCV/HIV coinfection in Brazil. In 2016, in a systematic review, Tengan et al estimated the seroprevalence of HCV among people living with HIV in Brazil. According to their data the seroprevalence of HCV in this population was ∼18.9%.^11^ Brazil has ∼700,000 people with chronic HCV infection and ∼25,000 cases of people living with HIV/AIDS have been identified every year since 2007.^12,13^ In Brazil, the pattern of HIV transmission has changed in recent years.^13^ The use of IDU as a transmission mechanism for this virus has decreased from 6.2% to 1.4% in males and from 2.2% to 1.2% in females, between 2007 and 2019.^13^ It is plausible that such changes in the pattern of HIV transmission could also impact the mode of HCV acquisition in this population.

This study sought to determine the prevalence of HCV infection in a cohort of HIV-infected individuals seen at a reference center in the city of Sao Paulo, Brazil, describe the demographic and clinical characteristics of this population, and delineate changes in the modes of HCV acquisition and the HCV genotype distribution in this cohort for the past 20 years.

## Materials and Methods

### Study design

Cross-sectional observational epidemiological study with data collected retrospectively from a patient cohort evaluated between January and December 2017.

### Study population

The individuals analyzed were those registered at the AIDS Outpatient Clinic of the Division of Infectious Diseases, at Hospital das Clínicas, School of Medicine at the University of São Paulo.

### Hepatitis C definition

HCV infection was defined as the detection of anti-HCV antibody and HCV RNA using commercial tests registered and approved by the Brazilian Ministry of Health. The HCV genotype was determined using direct sequence analysis of the 5′ noncoding region with the VERSANT HCV Genotype 2.0 assay (Siemens Healthcare Diagnostics Division, Tarrytown, NY, USA).

### Data collection

Participants were identified from a list of patients with an appointment scheduled between January and December 2017. All clinical and epidemiological data were extracted from an electronic database routinely prepared at the admission of the patient at the institution. Once the HCV-HIV coinfected cohort was identified, the database was utilized to obtain date of diagnosis of HCV infection, HCV genotype, sex, age, sexual orientation, and mechanisms of acquisition of HCV. The inclusion criterion was HCV/HIV coinfected individuals who had a scheduled appointment at the AIDS Outpatient Clinic between January 2017 and December 2017. The exclusion criterion was any patient with missing information in their charts.

The probable date of HCV acquisition was defined as the date of the first positive HCV serology in the medical record. For individuals with a history of blood transfusion, the probable date of infection was before 1993, the year in which blood banks began to strictly test for HCV. For individuals with a history of illicit IDUs, the probable date of infection was the individual's first reported use of illicit IDUs.^14^

When individuals reported more than one possible mode of HCV acquisition, we chose to define a prioritization order among them, according to biological plausibility.^15,16^ Thus, injecting drug use always prevailed over a history of blood transfusion or sexual transmission. Individuals were considered to have had a sexual exposure acquisition only if they denied IDU and blood transfusions. Acquisition by sexual exposure was defined when at least one of the following conditions was present: MSM, history of multiple sexual partners, or history of sexual relationship with a partner infected with HIV or HCV.

Dates of HCV infection were divided into three time periods: before 2000, between 2000 and 2009, and from 2010 onward. The period before 2000 was chosen since it represented the time when HCV infection in MSM HIV patients was not yet prominent. The other two time periods were chosen to allow for any progression or diminution in modes of exposure to be identified. Before the year 2000, patient group encompassed subjects evaluated from 1985 to 1999.

### Ethics

The study was approved by the local ethics committee (Comissão de Ética para Análise de Projetos de Pesquisa do HC FMUSP -Cappesq, Protocol No. CAAE: 37392414.5.0000.0068). Written informed consent from participants was deemed not to be necessary.

### Data analysis

Continuous variables were analyzed as mean ± standard deviation (SD) and categorical variables as absolute frequency (*n*) and percentage (%). For comparison between groups, chi-square or Fisher's exact tests were used, as appropriate. For analysis of proportions over time, the chi-square test for trends was used. The 5% statistical significance level was adopted. Data analysis was performed using STATA software version 15.1 (StataCorp, USA).

## Results

Between January and December 2017, 3,222 patients had a scheduled appointment at the AIDS Outpatient Clinic. Of these, 79 patients were excluded due to lack of clinical data. Thus, 3,143 patients were included in the study. The prevalence of HCV-HIV coinfection was 11.5% (362 individuals), 70.7% of whom were males. The mean age of patients in relation to the probable date of infection was 37 years (±11 years).

Overall, the reported modes of HCV acquisition were as follows: sexual exposure in 172 (47.5%), IDU in 86 (23.8%), use of inhaled drugs in 67 (18.5%), and history of blood transfusion in 10 (2.8%) individuals.

Regarding sexual orientation, 30 (8.2%) were bisexual, 87 (24%) homosexual, 236 (65.1%) heterosexual, and in 9 (2.4%) the data were absent from the medical records.

Table 1 presents the mechanisms of acquisition of HCV infection among HCV-HIV coinfected patients, grouped by time periods when transmission most likely occurred. Of those whose acquisition was by IDU, 84 (97.6%) occurred before 2000; the remaining two IDU subjects were infected between 2000 and 2009. In patients who acquired HCV by sexual exposure, 43 (25%) was before 2000, 62 (36%) between 2000 and 2009 and 67 (39%) from 2010 onward. Of the 105 individuals identified in the period from 2010 onward, the only mechanisms identified were the use of inhaled drugs in 27 (25.7%) and sexual exposure in 67 (63.8%). In 11 (10.5%) individuals, it was not possible to identify the mechanism of exposure from the medical records.

**Table 1.** Mechanisms of Hepatitis C Virus Acquisition Among HIV-Infected Individuals Over Time

Mechanism	<Year 2000 (*N* = 163),* n *(%)	Years 2000–2009 (*N* = 94),* n *(%)	>Year 2010 (*N* = 105),* n *(%)
Intravenous drugs	84 (51.5)	2 (2.1)	0 (0)
Transfusion	10 (6.1)	0 (0)	0 (0)
Inhaled drugs	20 (12.3)	20 (21.3)	27 (25.7)
Sexual exposure	43 (26.4)^a^	62 (65.9)^b^	67 (63.8)
Unavailable	6 (3.7)	10 (10.6)	11 (10.5)

^a^ *p* < .001 (<2000 vs. between 2000 and 2009).

^b^ *p* = .750 (between 2000 and 2009 vs. >2010).

There was an increase (*p* < .001) in the proportion of cases due to sexual transmission from the period <2000 (26.4%) to between 2000 and 2009 (65.9%). There was no corresponding increase from the period in between 2000 and 2009 and the subsequent period of >2010 (*p* = .751).

Table 2 shows the combined exposure mechanisms reported by patients by time period. In most of the cases sexual exposure occurred, even if it was not considered the main mechanism of infection. Of 362 individuals, 138 (38.1%) had more than one exposure mechanism. The combined mechanism of inhaled drugs and sexual exposure increased from the first period to the second one (*p* = .09) but remained stable from the second to the last one (*p* = .880), similar to the pattern observed for those that reported only the sexual exposure mechanism.

**Table 2.** Combined Mechanisms of Hepatitis C Virus Acquisition Among HIV-Infected Individuals Grouped by Time Periods

Combined mechanisms	<2000 (%) (*N* = 163)	2000–2009 (%) (*N* = 94)	>2010 (%) (*N* = 105)	Total (*N* = 362)
Sexual exposure	43 (26.4)	62 (66.0)	67 (63.8)	172 (47.5)
Inhaled drugs/sexual exposure	19 (11.7)^a^	18 (19.1)^b^	21 (20.0)	58 (16.0)
IDU/inhaled drugs/sexual exposure	43 (26.4)	1 (1.1)	0 (0)	44 (12.2)
Not known	6 (3.7)	10 (10.5)	11 (10.5)	27 (7.5)
IDU/inhaled drugs	18 (11.0)	1 (1.1)	0 (0)	19 (5.2)
IDU	11 (6.8)	0 (0)	0 (0)	11 (3)
IDU/sexual exposure	11 (6.8)	0 (0)	0 (0)	11 (3)
Inhaled drugs	1 (0.6)	2 (2.1)	6 (5.7)	9 (2.4)
Transfusion	5 (3.0)	0 (0)	0 (0)	5 (1.4)
Transfusion/sexual exposure	3 (1.8)	0 (0)	0 (0)	3 (0.8)
Transfusion/inhaled drugs/sexual	2 (1.2)	0 (0)	0 (0)	2 (0.6)
IDU/transfusion/inhaled drugs/sexual	1 (0.6)	0 (0)	0 (0)	1 (0.3)

^a^ *p* < .09 (<2000 vs. between 2000 and 2009).

^b^ *p* = .880 (between 2000 and 2009 vs. >2010).

Table 3 shows the HCV genotype distribution over time. Genotype 1 was most prevalent in each time period. The prevalence of genotype 3 decreased over time (test for trend *p* < .001). Genotype 4 was practically nonexistent in the first two time periods and then became the second most prevalent genotype from 2010 onward. There was an increase in cases that did not have genotyping results over time, from 28.2% in the first period to 39.4% in the second and 44.8% in the third (test for trend *p* = .008). Among the 130 patients with nonidentified genotypes, 106 were PCR-negative without a history of previous HCV treatment, 17 were PCR-negative with a prior specific HCV treatment, and genotype results were not available in seven patients.

**Table 3.** Hepatitis C Virus Genotype Distribution Among HCV-HIV Coinfected Patients, Grouped by Periods, São Paulo, 2017

HCV genotype	<2000 (%) (*N* = 163)	2000–2009 (%) (*N* = 94)	>2010 (%) (*N* = 105)	Total (*N* = 362)
1	80 (49.1)	46 (48.9)	40 (38.1)	166 (45.9)
2	3 (1.9)	1 (1.1)	0 (0)	4 (1.1)
3	32 (19.6)	10 (10.6)	4 (3.8)	46 (12.7)
4	2 (1.2)	0 (0)	14 (13.3)	16 (4.4)
Not available	46 (28.2)	37 (39.4)	47 (44.8)	130 (35.9)

HCV, hepatitis C virus.

The HCV genotype distribution according to exposure mechanisms, irrespective of time period is shown in Appendix Table A1. The genotype distribution in those who acquired HCV by sexual exposure was different from those who became HCV positive by IDU (*p* = .03). However, when comparing the genotype distribution for those that acquired HCV by sexual exposure with acquisition by all other mechanisms, the observed differences did not reach statistical significance (*p* = .121).

## Discussion

In this study, we assessed the rate and mechanisms of HCV acquisition as well as HCV genotype distribution in a cohort of HIV-positive individuals followed up for a 20-year time period. The prevalence of HCV coinfection in this cohort of HIV-positive individuals significantly decreased from 17.7% 20 years ago to 11.5% after 2010.^17^ This finding parallels data from the Ministry of Health regarding the decrease in IDU-induced HIV transmission in Brazil for the past 12 years^18^ and in part highlights the effectiveness of public health campaigns to reduce the risk of contracting these infections.

An important factor to be considered is that large changes have taken place in Brazil's illicit drug market in the past decade, affecting the structure, profile, and modes of distribution of illicit drugs in this country. According to the Third National Survey of Alcohol and Drugs by Brazil's National Institute for Public Policy Research on Alcohol and Other Drugs (2019), marijuana is the most consumed illicit drug in Brazil followed by snorted or smoked cocaine (crack) among the general population 12–65 years old. Compared with other drugs, crack is cheap, readily available, very addictive, and highly marketable.^19^

Nowadays, the prevalence of intravenous (IV) drug use is relatively rare in Brazil, occurring in 0.4% of persons 12–65 years of age during their lifetime. In the past the IV route was a more prevalent mechanism of illicit drug consumption.^19^ Owing to shared mechanisms of transmission of HCV and HIV, it is plausible that these changes in the mechanism of illicit drug usage also had an impact on the prevalence and modes of transmission of both infections, in concert with an enhanced public health campaign to increase awareness of reducing potential exposure to these viruses.

In our study, the main mechanism of HCV acquisition among individuals living with HIV was by sexual exposure. This mode of HCV transmission has been reported in several studies worldwide from the 2000s onward, predominantly in HIV-positive MSM individuals. Our findings are in accordance with these different studies.

However, it is important to comment that the observed decrease in IDU-related HCV transmission (after 2000) might mask a constant level of HCV sexual transmission that may have occurred during all periods of observation in our cohort. HCV sexual transmission was reported in each time period. Before 2000, it occurred in 25% of all cases. It is reasonable to suppose that both the effectiveness of public campaigns in reducing the risk of transmission of infections and the changes observed in the pattern of distribution and usage of illicit drugs in Brazil may have contributed to the observed general decrease in HCV-infected individuals. These factors likely contributed to a relative rise in the proportion of HCV cases due to sexual transmission in our cohort.

It is also plausible that specific lifestyle practices of MSM contributed to the observed rise in HCV sexual transmission cases in this group as compared with the general population. High-risk practices among a subpopulation of these individuals, such as anal intercourse with an increased number of sexual partners, recreational drug use to enhance sexual pleasure along with the widespread use of smartphone Apps, and online “hooking-up” sites to further increase the availability of casual sexual encounters, could contribute for this situation.^6–10^ Intestinal mucosal epithelial cell damage and release of pro-inflammatory cytokines due to chronic HIV infection likely enhances HCV acquisition among MSM.^20^ Also, the concentration of HCV-specific T lymphocytes may be reduced in immunosuppressed HIV-infected individuals, contributing to an inadequate anti-HCV response.^20^

Regarding HCV genotype distribution, previous studies have associated HCV genotype 4 infection with sexual acquisition.^21,22^ The increased prevalence of genotype 4 from 2010 onward observed in our study is consistent with our finding of enhanced acquisition of HCV by sexual exposure during this time period. In contrast, the prevalence of genotype 3 decreased over time (test for trend *p* < .001). This is likely associated with the decline in IDU over time, as previous studies have associated HCV genotype 3 infection with this mode of transmission.^23–25^

We acknowledge limitations in our study. An observational study necessarily limits data acquisition to findings contained in the medical record and may be incomplete. Also, as in all retrospective studies, recall bias may have had an impact on the ability of included individuals to accurately remember prior exposures. In addition, this investigation utilized files from only a single reference center. Thus, the observed findings may not be reflective of parameters of individuals in centers in other regions of Brazil and elsewhere.

However, these limitations are typical of this type of investigation, and we believe they do not negate the value and relevance of the results presented. Our study utilized a large cohort of HIV and HCV coinfected individuals and, as such, provides novel and relevant information on HCV transmission in Brazil. The Brazilian Ministry of Health, in line with the goal of the WHO to eliminate HCV as a global health threat by 2030, has outlined a national strategy to achieve this objective. Our findings are of value in re-evaluating public health strategies to minimize HCV acquisition and transmission in populations living with HIV/AIDS.

## Conclusions

Changes in the frequency of mechanisms of HCV acquisition in individuals living with HIV in Sao Paulo, Brazil for the past 20 years highlight that sexual exposure is now the major means of HCV transmission in this population.

## Appendix

**Appendix Table A1.** Hepatitis C Virus Genotypes in HCV-HIV Coinfected Individuals, Grouped by Mechanisms of Hepatitis C Virus Acquisition

Genotype	IDU*^a ^*(*N* = 62)	Transfusion (*N* = 8)	Inhaled drugs (*N* = 46)	Sexual exposure^b^ (*N* = 101)	Unknown (*N* = 15)	Total (*N* = 217)
1	41 (66.1)	7 (87.5)	33 (71.7)	72 (71.3)	13 (86.7)	153 (70.5)
2	2 (3.2)	0 (0)	0 (0)	2 (2)	0 (0)	4 (1.8)
3	18 (29)	1 (12.5)	9 (19.6)	16 (15.8)	2 (13.3)	44 (20.3)
4	1 (1.6)	0 (0)	4 (8.7)	11 (10.9)	0 (0)	16 (7.3)

^a^ *p* < .03 (IDU vs. sexual exposure).

^b^ *p* = .121 (sexual vs. nonsexual exposure).

IDU, injection drug use.
